# Disability and functional outcomes following STN, VC/VS, and combined deep brain stimulation in obsessive compulsive disorder

**DOI:** 10.1192/bjo.2021.790

**Published:** 2021-06-18

**Authors:** Himanshu Tyagi

**Affiliations:** UCL Institute of Neurology, Queen Square, The National Hospital for Neurology and Neurosurgery

## Abstract

**Background:**

OCD severity scores mostly convey information within the domain of clinical conceptualisations. To capture the full impact of any new intervention it is crucial to measure its impact on disability. For this purpose we captured prospective data on changes in disability, function and impairments with multiple tools throughout the UCL-MRC trial of Deep brain stimulation (DBS) for Obsessive Compulsive Disorder (OCD) between 2013-2017. The clinical and cognitive outcomes from the trial have already been reported in 2019. We hypothesized a concomitant improvement in perceived and observed indicators of disability with clinical improvement in OCD symptoms. This is a preliminary report of the disability outcome data from the trial.

**Method:**

Six patients with severe treatment resistant OCD were recruited for this study from the NHS England OCD Specialist Service. Eligible participants were offered lesion surgery (anterior cingulotomy) or entry to the DBS trial. OCD medication was kept constant throughout the trial. We tested the effects of DBS by comparing baseline, VC/VS, STN, both sites and CBT stages of trial on the following three assessments of function: GAF, SDS and CAOIC. An impairment focussed interview was done to quantify changes in function specific to OCD. Friedman's test was used to test for DBS effects during the double-blind crossover phases comparing baseline, amSTN and VC/VS. Post-hoc pair-wise Conover tests for significant effects were used with FDR corrections. All significant results are reported at P < 0.05.

**Result:**

DBS had a significant effect across all phases for all above mentioned clinical measures. For all three measures of disability, there were significant improvements after both amSTN and VC/VS DBS. For all three measures of disability the effect of VC/VS DBS was significantly better that amSTN DBS. For all three measures of disability there was a non-significant trend (p = 0.058) for stimulation at both sites to have a better effect than stimulation of one site alone. For all three measures of disability, there were no significant difference between DBS alone and DBS and CBT.

**Conclusion:**

This study is the first to have directly compared differential effects of STN versus VC/VS DBS stimulation in OCD patients whilst testing clinical, cognitive and disability outcomes. The results of this study indicate that although both sites are equally effective in reducing OCD, stimulating VC/VS leads to a significantly greater improvement on disability scores in severe OCD.

